# Effects of secular changes in tidal volume and respiratory rate on the mechanical power of ventilation: a retrospective single-center study of invasively ventilated patients

**DOI:** 10.62675/2965-2774.20250403

**Published:** 2025-09-11

**Authors:** Abdelrahman Mahmoud M. Senosy, Charalampos Pierrakos, Ary Serpa, Marcus J. Schultz

**Affiliations:** 1 Hayat National Hospital Department of Intensive Care Unit Madina Saudi Arabia Department of Intensive Care Unit, Hayat National Hospital - Madina, Saudi Arabia.; 2 Université Libre de Bruxelles Brugmann University Hospital Department of Intensive Care Brussels Belgium Department of Intensive Care, Brugmann University Hospital, Université Libre de Bruxelles - Brussels, Belgium.; 3 Monash University School of Public Health and Preventive Medicine Australian and New Zealand Intensive Care Research Centre Melbourne Australia Australian and New Zealand Intensive Care Research Centre, School of Public Health and Preventive Medicine, Monash University - Melbourne, Australia.; 4 Amsterdam University Medical Centers Department of Intensive Care and Laboratory of Experimental Intensive Care and Anesthesiology Amsterdam Netherlands Department of Intensive Care and Laboratory of Experimental Intensive Care and Anesthesiology, Amsterdam University Medical Centers - Amsterdam, The Netherlands.

**Keywords:** Intensive care, Ventilation, Tidal volume, Respiratory rate, Intensity of ventilation, Mechanical power

## Abstract

**Objective:**

To evaluate how secular changes in tidal volume and respiratory rate influence the mechanical power of ventilation during the first 24 hours in critically ill patients over two decades and to compare their effects in patients with high and low respiratory system compliance.

**Methods:**

This secondary analysis of the Amsterdam University Medical Center database included two time periods: 2003 to 2009 and 2010 to 2016. The primary endpoint was mechanical power. Analyses also assessed secular changes in mechanical power in patients with respiratory system compliance groups.

**Results:**

Among 4,877 patients (2,536 patients in 2003 - 2009, and 2,341 in 2010 - 2016), median tidal volume decreased (mean difference of −0.6 [-0.4 to −0.7] mL/kg predicted body weight; p < 0.01), median respiratory rate increased (mean difference of +1.0 [+0.75 to +1.25] breath/minute; p < 0.01), and median mechanical power fell from 12.1 (8.7 - 16.7) J/minute to 10.4 (7.6 - 14.6) J/minute (mean difference of −1.7 [-1.2 to −2.0] J/minute; p < 0.01). In patients with low respiratory system compliance, median mechanical power decreased more significantly (13.4 J/minute to 11.7 J/minute, mean difference of −1.7 J/minute; p < 0.01) compared to those with high respiratory system compliance (10.5 J/minute to 9.7 J/minute, mean difference of −0.8 J/minute; p < 0.01) despite comparable respiratory rate changes

**Conclusion:**

In this single-center cohort, secular changes in tidal volume and respiratory rate were associated with lower mechanical power, particularly in patients with low respiratory system compliance.

## INTRODUCTION

Ventilation with a low tidal volume (V_T_) has been shown to improve outcomes of patients with acute respiratory distress syndrome (ARDS) compared to ventilation with a high V_T_.^( 1 )^ Consequently, clinical practices have shifted towards ‘low V_T_ ventilation’ (LTVV), not only in patients with ARDS but also in patients not having this severe form of acute hypoxemic respiratory failure.^( 2 - 6 )^ We may want to refine the concept that V_T_ should invariably be low. Firstly, we have gained insights into the significance of a low driving pressure (ΔP), which may be equally critical.^( 7 )^ Secondly, adopting LTVV can lead to a compensatory increase in respiratory rate (RR) to maintain minute ventilation with potential consequences.^( 8 , 9 )^ Last but not least, the interplay between these key ventilator settings could be crucial, as a combination of low V_T_ with high RR could be beneficial in a patient with a lower respiratory system compliance (C_RS_), but harmful in patients with higher C_RS_.^( 10 , 11 )^

Rather than focusing solely on V_T_, mechanical power (MP) of ventilation integrates V_T_, airway pressures, and RR to quantify the total energy delivered to the respiratory system.^( 12 , 13 )^ Although MP equations assume a direct relationship between V_T_ and MP, the physiological interplay is more complex. Changes in RR may compensate for reductions in V_T_ to maintain minute ventilation, while both V_T_ and RR can affect airway pressures in different ways.^( 14 )^ Despite its physiological relevance, MP remains underutilized in clinical practice, as LTVV is simpler to implement and does not require complex calculations.^( 13 )^ However, whether reducing V_T_ alone reliably minimizes mechanical energy exposure in clinical settings remains uncertain.

Our objective was to evaluate how secular changes in V_T_ and RR influence the mechanical power of ventilation during the first 24 hours in critically ill patients over two decades and to compare their effects in patients with high and low respiratory system compliance. We assessed how secular changes in V_T_ and RR affect MP in invasively ventilated patients in a large single-center cohort spanning from 2003 to 2016. We hypothesized that a more intense use of LTVV would decrease MP. Additionally, we compared the effects of secular changes in V_T_ and RR on MP in patients with high *versus* low C_RS_.

## METHODS

### Design and setting

This is a secondary analysis of the Amsterdam University Medical Center database (the AmsterdamUMCdb; version 1.0.2, released March 2020). AmsterdamUMCdb is a publicly freely accessible database, comprising anonymized health data from intensive care unit (ICU) patients between 2003 and 2016. The creation of this database was endorsed by the European Society of Intensive Care Medicine (ESICM) and its Data Science Section. An external privacy expert audited the process, and the Ethics Section of the ESICM provided external ethics review and appraisal. Need for individual patient consent was waived as this database collected data captured as part of routine care, and data were deidentified to protect patient privacy. Compliance with General Data Protection Regulation (GDPR) and Health Insurance Portability and Accountability Act (HIPAA) was ensured through detailed legal reviews and robust privacy safeguards, overcoming legal and ethical hurdles.^( 15 )^

### Inclusion and exclusion criteria

Patients were eligible for this secondary analysis if they were aged over 16 years and they had received invasive ventilation for at least 24 hours. Patients were excluded in case their datasets were incomplete about ventilator variables and parameters necessary for the calculation of MP. In case a patient was admitted more than once, only the data of the first admission were used.

### Data collected

Baseline demographics and patients’ characteristics were captured at baseline. The severity of patients was evaluated based on the Acute Physiology and Chronic Health Evaluation (APACHE II) and the Sequential Organ Failure Assessment (SOFA) score. Ventilatory variables, including V_T_, airway pressures, and RR, were recorded at a frequency of up to one value per minute following patient intubation. To create a manageable dataset, we calculated the average value of each variable for blocks of 60 minutes, thereby reducing the dataset to a single averaged value per variable for each time block.

### Calculations

Tidal volume was expressed in mL/kg predicted body weight (PBW), and ΔP, C_RS_, and MP were calculated using the following equations:^( 1 )^


[Eq. 1] PBW=45.5+0.91∗(centimeters of height−152.4)(for females)

and


[Eq. 2] PBW=50+0.91* (centimeters of height−152.4)(for males)

The dynamic MP was calculated using the following equation:


[Eq. 3]^( 12 , 16 , 17 )^ MP(J/minute)=0.098∗VT(L)∗RR∗(Pmax−12∗ΔP)

Driving pressure and C_RS_ were calculated using the following equations:


[Eq. 4] $$\text{ΔP}\left({\text{cmH}}_{2}\text{O}\right)=\text{Pmax-positive end-expiratory pressure (PEEP)}$$

and


[Eq. 5] $${\text{C}}_{\text{RS}}\left(\text{mL}/{\text{cm H}}_{2}\text{O}\right)={\text{V}}_{\text{T}}(\text{mL})/\text{ΔP}\left({\text{cmH}}_{2}\text{O}\right)$$

### Definitions

Low V_T_ ventilation was defined as ventilation with V_T_ < 8mL/kg PBW.^( 6 )^ Low-intensity was defined pragmatically as ventilation with ΔP < 15cmH_2_O^( 7 )^ and MP < 17J/minute,^( 17 )^ while high-intensity ventilation was defined as ΔP ≥ 15cmH_2_O or MP ≥ 17J/minute. Accordingly, groups were labeled as follows: ventilation with low V_T_ at low-intensity (LV-L), ventilation with high V_T_ at low-intensity (HV-L), ventilation with low V_T_ at high-intensity (LV-H), and ventilation with high V_T_ at high-intensity (HV-H). We used the median split method to create the two subgroups, low C_RS_ and high C_RS_.^( 18 )^

### Study endpoints

Mechanical power, calculated 24 hours after the initiation of invasive ventilation, was the primary endpoint of this analysis. Secondary exploratory endpoints included ICU-, 28-, and 90-day mortality, ICU length of stay, and ventilator-free days in ICU survivors at day 28 (VFD-28).

### Sample size

We did not perform a formal sample size calculation; instead, the number of eligible patients in the database served as the sample size.

### Statistical analysis

Demographic, clinical, and outcome variables were presented as percentages for categorical variables and as medians with interquartile ranges (IQR) for continuous variables.

We anticipated a shift toward lower V_T_ in the second temporal cohort of patients, following a broader implementation to patients without ARDS.^( 19 - 21 )^ For analytical purposes, the AmsterdamUMCdb was divided into two cohorts, one lasting from 2003 to 2009 and one from 2010 to 2016. We employed time-weighted averaging for each variable to account for daily fluctuations in the data. The daily value of each variable for the patient was calculated by averaging the medians of four 6-hour intervals over the first 24 hours. To compare variables between patients in the two temporal cohorts, we performed the Wilcoxon signed-rank test for continuous variables and Fisher's exact test for categorical variables.

We used cumulative distribution plots to visualize ventilator settings and MP differences between the two temporal cohorts. Herein, V_T_, ΔP, RR, and MP were plotted against cumulative frequencies ranging from 0 to 1. Both temporal cohorts were plotted on the same graph to facilitate comparison, providing a visual representation of differences in ventilator variables and parameters. Tidal volume, ΔP, RR, and MP correlations are visualized using scatter plot diagrams.

The cumulative incidence of ICU discharge and mortality over time was calculated based on the type of ventilation: LV-L, LV-H, HV-L, and HV-H. Discharge and death were treated as competing events, with prolonged ICU hospitalization serving as the reference. The follow-up period continued until the end of the study, at which point the data were censored. Ventilation-free days up to 28 days were calculated by subtracting the total number of days a patient was on mechanical ventilation within the first 28 days from 28.

We performed several post hoc analysis. Since we noticed a difference in patient demographics between the two secular cohorts - a 10% higher proportion of medical patients in the 2010 - 2016 cohort - to address this issue, we conducted a post hoc analysis in which we first divided the cohort into medical and surgical patients, and then repeated the analysis within each group separately. In a second post hoc analysis, we employed a simplified mechanical power equation specifically adapted for pressure-controlled ventilation modes ( Table 1S - Supplementary Material ).^( 22 )^ In a third post hoc analysis, we calculated MP using alternative equations ( Table 1S - Supplementary Material ), and we normalized MP to PBW and to C_RS_ as done previously.^( 9 , 23 , 24 )^

All analyses were performed in R through the R-studio interface (www.r-project.org, R version 4.3.1). A p-value < 0.05 was considered significant.

## RESULTS

### Patients

**Figure 1:**
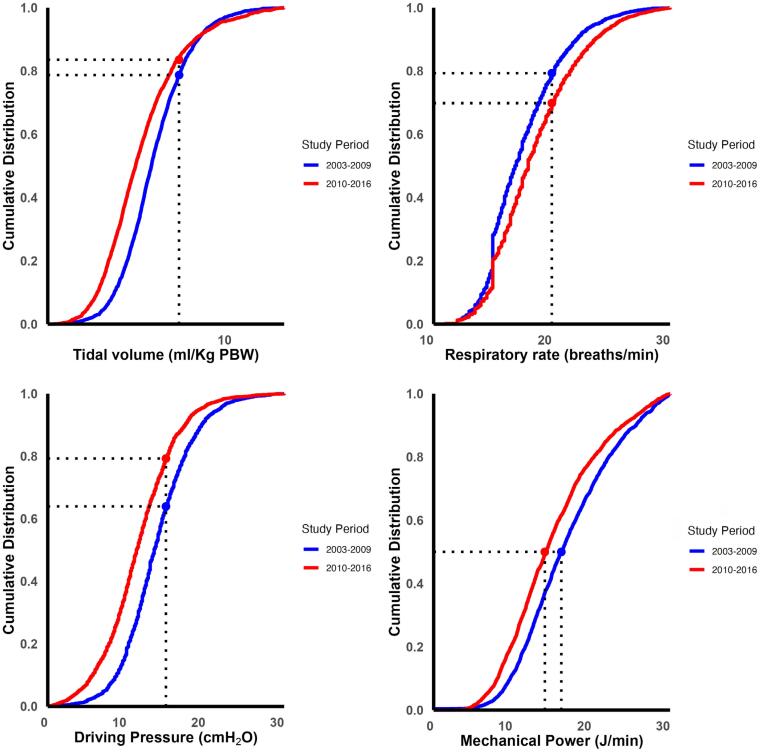
Cumulative distribution curves for tidal volume, respiratory rate, driving pressure and mechanical ventilation power comparing two study periods, from 2003 to 2009 and from 2010 to 2016.

Of the 4,877 included patients, 2,536 were admitted between 2003 and 2009 and 2,341 between 2010 and 2016 ( Figure 1S - Supplementary Material ). Baseline demographics and patient characteristics were similar for the two temporal cohorts ( Table 1 ). Patients with low C_RS_ had a higher prevalence of females, ICU hospitalizations mostly related to respiratory problems, and lower oxygenation metrics compared to those with high C_RS_.

**Table 1:** Characteristics of patients from 2003 - 2009 and 2010 - 2016, shown for both the total population and subgroups categorized by low and high respiratory system compliance, with a cut-off value of 36mL/cmH_2_O

Baseline characteristics Total population		2003 - 2009			2010 - 2016		
		Low C_RS_	High C_RS_	Total population	Low C_RS_	High C_RS_	
Number of patients		2,536	1,489	744	2,341	1,274	1025
Female (gender)		883 (35)	528 (45)	254 (34)	805 (34)	432 (44)	352 (34)
Age of patients		65 (55 - 75)	64 (54 - 74)	64 (54 - 74)	65 (55 - 75)	64 (54 - 74)	64 (54 - 74)
Weight (kg)		75 (65 - 85)	74 (64 - 84)	75 (74 - 84)	75 (65 - 85)	74 (64 - 84)	84 (74 - 84)
Height (cm)		175 (165 - 185)	174 (164 - 184)	174 (174 - 184)	175 (165 - 185)	174 (164-184)	174 (174 - 184)
Predicted body weight (kg)		70 (61 - 79)	70 (61 - 79)	70 (65 - 79)	70 (61 - 79)	70 (61 - 79)	70 (65 - 79)
Low tidal volume ventilation (%)		1,758 (78)	1,310 (88)	448 (60)	1,921 (82)	1,243 (97)	678 (66)
Admission type							
	Surgical elective (%)	696 (27)	416 (28)	193 (26)	387 (17)	172 (14)	208 (20)
	Surgical urgency (%)	542 (21)	282 (20)	163 (22)	514 (22)	253 (20)	257 (25)
	Medical (%)	1298 (51)	1175 (53)	386 (52)	1439 (61)	848 (66)	560 (55)
Need for support for the first 24 hours							
	Vasopressors (%)	2,283 (90)	1,394 (94)	643 (89)	2,126 (90)	1,208 (95)	878 (86)
	Renal replacement therapy (%)	411 (16)	296 (20)	71 (9)	352 (15)	214 (17)	122 (12)
Initial diagnosis							
	Sepsis (%)	464 (18)	297 (20)	112 (15)	391 (17)	226 (18)	155 (15)
	Cardiovascular disease (%)	708 (28)	416(28)	201 (27)	638 (27)	430 (34)	193 (19)
	Respiratory condition (%)	265 (10)	148 (10)	89 (12)	338 (14)	186 (15)	146 (15)
	Neurological condition (%)	222 (9)	150 (10)	59 (8)	304 (13)	128 (10)	172 (17)
	Trauma (%)	66 (3)	28 (2)	30 (4)	170 (7)	80 (6)	89 (9)
	Other (%)	811 (32)	446 (30)	252 (33)	499 (21)	195 (15)	245 (25)
Severity of disease							
	APACHE II score	21 (17 - 26)	22 (18 - 26)	21 (17 - 25)	23 (19 - 28)	24 (19 - 30)	22 (18 - 26)
	SOFA score	9 (7 - 11)	9 (7 - 11)	9 (7 - 11)	9 (7 - 11)	10 (7 - 12)	9 (7 - 11)
Vital signs							
	P_a_O_2_/FiO_2_ (mmHg)	147(96 - 207	138 (88 - 192)	168 (110 - 226)	145 (97 - 210)	136 (90 - 195)	156 (108 - 232)
	Heart rate (beats/minute)	106 (91 - 124)	107 (91 - 126)	105 (90 - 122)	116 (98 - 135)	112 (94 - 133)	119 (103 - 137)
	Mean arterial pressure (mmHg)	60 (53 - 66)	59 (53 - 65)	60 (54 - 67)	52 (38 - 61)	53 (40 - 61)	50 (37 - 61)
	Temperature (degrees Celsius)	37 (36 - 37)	36 (35 - 37)	36 (35 - 37)	37 (36 - 37)	35 (35 - 36)	36 (35 - 37)
Laboratory data							
	pH	7.29 (7.21 - 7.34)	7.33 (7.25 - 7.40)	7.37 (7.32 - 7.42)	7.27 (7.19 - 7.33)	7.31 (7.23 - 7.38)	7.36 (7.29 - 7.41)
	PaCO_2_ (mmHg)		40 (36 - 44)			41 (37 - 46)	
	CRP (mg/L)	63 (11 - 149)	66 (12 - 158)	62 (11 - 145)	55 (8 - 140)	55 (8-140)	57 (8 - 148)
Outcomes							
	Ventilation-free days	17 (2 - 23)	16 (1 - 23)	21 (10 - 25)	20 (4 - 25)	17 (1 - 23)	23 (12 - 26)
	ICU mortality (%)	523 (21)	337 (23)	108 (15)	571 (24)	360 (28)	181 (18)

Crs - respiratory system compliance; APACHE II - Acute Physiology and Chronic Health Evaluation II; SOFA - Sequential Organ Failure Assessment; PaO_2_ - partial pressure of arterial oxygen; FiO_2_ - fraction of inspired oxygen; PaCO_2_ - partial pressure of arterial carbon dioxide; CRP - C-reactive protein; ICU - intensive care unit. Results are presented as medians with interquartile ranges or percentages where appropriate.

### Secular changes in tidal volume, respiratory rate, and mechanical power

**Figure 2:**
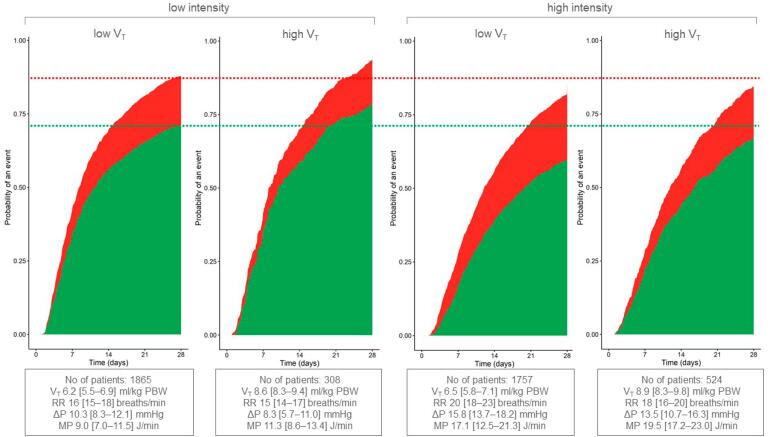
Cumulative incidence of intensive care unit discharge and mortality within 28 days by ventilation intensity and tidal volume per predicted body weight.

From 2003 to 2009 to 2010 to 2016, median V_T_ decreased (mean difference [MD] of −0.6 [-0.4 to −0.7] mL/kg PBW; p < 0.01) and median RR increased (MD of +1.0 [+0.75 to +1.25] breath/minute; p < 0.01) ( Figure 1 and Table 2 , and Table 1S and Figure 2S [Supplementary Material] ). The percentage of patients who received LTVV increased from 76% to 82%. The temporal changes in V_T_ and RR were associated with a decrease in median MP from 12.1 (8.7 to 16.7) J/minute to 10.4 (7.6 - 14.6) J/minute (MD −1.7 [-2.0 to −1.2] J/minute; p < 0.01). The percentage of patients with MP of < 17 J/minute increased from 76 to 83%.

V_T_ - tidal volume; PBW - predicted body weight.

The dotted lines indicate the cutoff values used to define lung-protective ventilation strategies, for tidal volume (8mL/kg of predicted body weight), driving pressure (15cmH_2_O), respiratory rate (20 breaths/minute), and mechanical power of ventilation (17J/minute).

**Table 2:** Ventilation parameters of patients from 2003 - 2009 and 2010 - 2016, shown for both the total population and subgroups categorized by low and high respiratory system compliance, with a cut-off value of 36mL/cmH_2_O

Variables		2003 - 2009			2010 - 2016		
		Total population	Low C_RS_	High C_RS_	Total population	Low C_RS_	High C_RS_
Compliance (mL/cmH_2_O)		34.9 (27.4 - 45.2)	30.1 (24.4 - 35.6)	50.2 (43.7 - 62.4)	38.1 (28.1 - 56.6)	29.2 (23.6 - 35.2)	60.8 (48.9 - 89.5)
Tidal volume (mL)		456 (422 - 536)	456 (410 - 511)	511 (459 - 586)	435 (378 - 511)	400 (353 - 449)	499 (430 - 594)
Tidal volume per PBW (mL/kg)		6.9 (6.1 - 7.8)	6.6 (5.9 - 7.3)	7.6 (6.8 - 8.6)	6.3 (5.5 - 7.4)	5.7 (5.1 - 6.4)	7.3 (6.4 - 8.5)
PEEP (cmH_2_O)		8.5 (6.2 - 10.5)	9.0 (6.8 - 11.5)	7.5 (5.7 - 9.2)	7.9 (5.8 - 9.9)	8.2 (6.2 - 10.7)	7.2 (5.3 - 9.0)
Respiratory rate (breaths/minute)		17.1 (15.2 - 19.5)	18.0 (15.6 - 20.5)	15.5 (14.8 - 17.3)	18.2 (15.7 - 20.7)	18.8 (16.6 - 22.5)	16.8 (15.0 - 19.2)
Driving pressure (cmH_2_O)		13.5 (11.0 - 16.6)	15.2 (13.2 - 17.8)	11.1 (9.2 - 12.5)	11.2 (8.5 - 14.5)	13.8 (11.8 - 16.0)	8.2 (6.0 - 10.0)
MP_dyn_ (J/minute)		12.1 (8.7 - 16.7)	13.4 (9.7 - 19.1)	10.5 (8.1 - 14.5)	11.2 (8.5 - 14.6)	11.7 (8.2 - 16.3)	9.7 (7.2 - 13.3)
MP_PC_		17.4 (12.8 - 23.9)	19.9 (14.7 - 26.8)	13.7 (10.5 - 17.9)	14.8 (10.8 - 20.7)	16.8 (12.3 - 22.5)	12.6 (9.1 - 17.4)
MP_NORM_ (J/minute/kg per PBW)		257 (191 - 352)	288 (215 - 392)	206 (158 - 273)	217 (156 - 298)	244 (180 - 329)	188 (133 - 257)
MP_CRS_ (J/minute × cmH_2_O/mL)		496 (314 - 795)	657 (461 - 997)	271 (195 - 372)	383 (225 - 641)	574 (393 - 879)	211 (128 - 309)
MP_CRS-NORM_ (J/minute x cmH_2_0/mL /kg per PBW)		7.2 (4.5 - 11.9)	9.5 (6.6 - 14.6)	4.1 (2.8 - 5.8)	5.5 (3.2 - 9.4)	8.3 (5.5 - 13.2)	3.1 (1.8 - 4.7)
Dynamic power (J/minute)		10.7 (7.8 - 14.5)	12.3 (9.4 - 16.3)	7.8 (6.1 - 10.1)	8.6 (6.1 - 12.1)	10.2 (7.8 - 13.8)	6.6 (4.5 - 9.2)
Costa index		71 (60 - 85)	79 (70 - 91)	56 (49 - 63)	63 (52 - 77)	74 (65 - 86)	50 (41 - 58)
Type of ventilation							
	Number of patients	2,209	1,484	604	2,245	1,262	983
	Low Low tidal volume - Low intensity (%)	769 (35)	399 (27)	370 (51)	1,096 (49)	535 (42)	561 (57)
	Low tidal volume - High intensity (%)	974 (44)	906 (61)	68 (10)	783 (35)	696 (55)	87 (9)
	High tidal volume - Low intensity (%)	149 (7)	9 (1)	140 (19)	159 (7)	0 (0)	159 (16)
	High tidal volume - High intensity (%)	317 (14)	170 (11)	147 (20)	207 (9)	31 (3)	176 (18)

C_RS_ - respiratory system compliance; PBW - predicted body weight; PEEP - positive end-expiratory pressure; MP_dyn_ - dynamic mechanical power; MP_PC_ - mechanical power adapted for pressure control ventilation mode; MP_NORM_ - dynamic mechanical power normalized to predicted body weight; MP_CRS_ - dynamic mechanical power normalized to respiratory system compliance; MP_CRS-NORM_: - dynamic mechanical power normalized to respiratory system compliance and predicted body weight. Results are presented as medians with interquartile ranges or percentages where appropriate.

### Secular changes in high versus low C_RS_ patients

Whilst there was a clear difference in the decrease in V_T_ between the two C_RS_ groups, with a larger reduction in median V_T_ in patients with low C_RS_, the changes in median RR were comparable between the two C_RS_ groups ( Figure 1 and Table 2 , and Table 1S and Figure 2S [Supplementary Material] ). The reduction in median MP was less pronounced in patients with high C_RS_ (MD of −0.81 J/minute [-1.31 to −0.44]) compared to patients with low C_RS_ (MD −1.68 J/minute [-2.28 to −1.22]).

### Association of tidal volume and ventilation intensity with intensive care unit mortality in the two temporary cohorts

Independent of V_T_, high-intensity ventilation (LV-H, HV-H) was associated with higher mortality compared to low-intensity ventilation (LV-L, HV-L). The combination of ventilation with higher V_T_ at low-intensity ventilation (HV-L) resulted in the lowest mortality rate ( Figure 2 , and Figure 3S [Supplementary Material] ). Also independent of V_T_, high-intensity ventilation resulted in fewer VFD-28 ( Figure 4S - Supplementary Material ). The differences in mortality based on ventilation intensity were more pronounced in patients with low C_RS_ than those with high C_RS_ ( Figures 5S and 6S - Supplementary Material ).

V_T_ - tidal volume; RR - respiratory rate; ΔP - driving pressure; MP - mechanical ventilation power.

The X-axis shows the days since intensive care unit admission, and the Y-axis shows the probabilities of intensive care unit discharge (green) and death (red). Left panels show groups at low-intensity ventilation, while right panels show groups at high-intensity ventilation, for patients ventilated with low and higher tidal volumes. The red line marks the highest cumulative incidence of any event (intensive care unit discharge or death). In contrast, the green line marks the highest intensive care unit discharge in patients receiving ventilation with low tidal volumes at low intensity. Key ventilatory parameters are presented as medians with interquartile range in the boxes under each graph.

### Post hoc analysis

Results from a post hoc analysis in which we repeated the analysis within medical and surgical patients separately are shown in tables 3S and 4S , and figures 7S and 8S (Supplementary Material) . The results were consistent with the primary analysis in both medical and surgical patients. Furthermore, no differing patterns emerged when using the pressure control-oriented MP formula or alternative methods for MP assessment ( Table 2 , Tables 3S and 4S [Supplementary Material] ).

## DISCUSSION

The main findings of this study can be summarized as follows: most patients received LTVV with median V_T_ declining over the years; despite a modest compensatory rise in RR, this shift resulted in an overall decrease in MP; the impact of lower V_T_ on MP varied with C_RS_, showing less pronounced effects in patients with high C_RS_; ventilation intensity was associated with increased mortality in patients ventilated with LTVV or high V_T_, particularly in patients with low C_RS_.

Our study has several strengths. The dataset is extensive, covering more than 13 years, which allows for a robust evaluation of changes in ventilatory practices over time. It includes a diverse patient population with a wide range of pathologies and V_T_ values, enhancing the generalizability of the findings. Additionally, the patients presented a broad spectrum of C_RS_, enabling a detailed analysis of how adjustments in V_T_ and RR affect MP across different respiratory mechanics subgroups. Furthermore, the study's design, which incorporates time-weighted averaging to account for daily fluctuations in ventilatory variables, provides a robust method of data analysis. This approach minimizes the risk of bias from transient changes and allows for a more accurate assessment of the effects of V_T_ on MP in real-life settings.

The results of this study align with previous research,^( 5 , 6 , 25 - 28 )^ consistently demonstrating a trend of decreasing V_T_ in patients receiving invasive ventilation over recent decades. Notably, the majority of patients received V_T_ below 8mL/kg PBW during both study periods, indicating that the LTVV strategy has been adopted early at this medical facility.^( 25 , 29 , 30 )^ The more pronounced decrease in V_T_ in patients with low CRS suggests that clinicians were particularly cautious in managing airway pressures. Clinicians actively lowered VT to the lowest feasible levels when airway pressure-derived parameters, such as plateau or driving pressures, were elevated - indicating increased strain on the lungs. This approach likely reflects an effort to minimize lung injury and reduce mechanical stress in patients with compromised respiratory mechanics, in which small adjustments in ventilation settings could significantly impact lung protection. Therefore, our study corroborates trends in V_T_ utilization in the context of LTVV and demonstrates a consistent effort to employ even smaller V_T_ to improve lung mechanics.

Previous research in ARDS patients did not show a secular change in RR with decreasing V_T_ over time, as only the highest RR values were considered in that study.^( 5 )^ In contrast, we reported time-weighted average RR values. Another study using time-weighted average RR also observed a reduction in V_T_ but without a corresponding increase in RR; however, V_T_ often exceeded 8mL/kg PBW in that study.^( 4 )^ Our findings suggest that in patients receiving LTVV, further reducing V_T_ lowers airway pressures and subsequently reduces MP, even if RR increases.

Our observations are consistent with findings from an experimental mathematical model in ARDS patients, which showed that reducing V_T_, even if it leads to a modest increase in RR, is an effective strategy for lowering MP.^( 31 )^ Indeed, our study suggests that excessively low V_T_ may not be advantageous per se in patients ventilated receiving LTVV. Although minimizing V_T_ increased the number of patients receiving lung protective ventilation, a notable number of patients received LTVV with high intensity, which may worsen outcomes. Therefore, the results of this study support the idea that the primary benefit of low V_T_ is the reduction in ΔP rather than the reduction in V_T_ itself, as indicated in a previous study.^( 32 )^

In patients with elevated C_RS_, the compensatory increase in RR was disproportionately greater than the decrease in V_T_, rendering V_T_ reduction less effective in lowering MP than patients with lower C_RS_. Although the precise rationale behind this discrepancy remains uncertain, it is conceivable that, in patients with reduced compliance, a certain degree of permissive hypercapnia was tolerated, whereas in those with higher compliance, maintaining minute ventilation was prioritized.^( 9 )^ Regardless of the underlying mechanisms, our findings reinforce the notion that increases in RR substantially amplify MP, aligning with prior observations.^( 33 )^ Moreover, these results emphasize the necessity of adapting ventilatory strategies to individual respiratory mechanics to optimize MP, thereby supporting modeling-based hypotheses suggesting that the impact of V_T_ reduction on clinical outcomes may differ according to baseline C_RS_.^( 9 )^

This study has limitations. The retrospective design limits the ability to establish causality between V_T_, RR, and MP trends with the outcome. Therefore, we cannot prove that changes in V_T_, RR, or MP directly influenced outcomes as unmeasured confounders may have influenced both ventilatory settings and clinical outcomes. Differences observed between the two secular cohorts may reflect changes in local hospital organization over time. Including all mechanically ventilated patients increases generalizability but introduces diagnostic heterogeneity. The post hoc analysis, in which we first divided the cohort into medical and surgical patients, and then repeated the analysis within each group separately, showed similar trends in V_T_, RR, and MP in both medical and surgical ICU admissions, indicating consistent practice changes across diagnostic groups. Additionally, as in previous research, MP was calculated based on dynamic variables.^( 16 , 17 )^ Although airway ΔP correlates with transpulmonary ΔP, it remains a surrogate that can be affected by factors like resistive airway pressures and chest wall compliance, which were not evaluated here. As a result, transpulmonary pressure could be underestimated, particularly in our population where V_T_ decreased substantially and RR increased, leading to only subtle changes in respiratory system pressure while promoting air trapping and potential auto-PEEP. The relevance of total MP has been challenged with special criticism towards its PEEP component^( 34 )^ and concerns regarding the ability of the proposed initially formula to accurately measure MP in patients receiving pressure-controlled ventilation.^( 35 )^ Nevertheless, the additional analyses using alternative equations for calculating MP also confirmed the findings of the primary analysis. Lastly, although our analysis was limited to the first 24 hours after intubation, when spontaneous breathing efforts are minimal, we could not reliably distinguish and exclude such efforts, potentially affecting the accuracy of driving pressure and MP estimates.

## CONCLUSION

In this single-center cohort of patients receiving invasive ventilation for at least 24 hours, secular changes in tidal volume were associated with a decline in median mechanical power, despite compensatory increases in respiratory rate. This association was less pronounced in patients with high respiratory system compliance, suggesting that the potential benefits of further tidal volume reduction warrant careful consideration within the framework of low tidal volume ventilation.

Data availability

The AmsterdamUMCdb dataset that we employed in our research is open-access and available upon request. To gain access to the database, the requester must complete a specific training course focusing on managing deidentified clinical data. Please visit https://amsterdammedicaldatascience.nl for additional information on how to access AmsterdamUMCdb.
