# Supplementary material for: Effects of secular changes in tidal volume and respiratory rate on the mechanical power of ventilation: a retrospective single-center study of invasively ventilated patients
Source: Crit Care Sci. 2025 Sep 11;37:e20250403. doi: 10.62675/2965-2774.20250403 (PMC12614958; doi:10.62675/2965-2774.20250403)
Supplement: Supplementary file 1 [file 2965-2774-ccsci-37-e20250403-Suppl01.pdf]

Critical Care Science
https://doi.org/10.62675/2965-2774.20250403

SUPPLEMENTARY MATERIAL

Effects of secular changes in tidal volume and respiratory rate on the mechanical power of ventilation: a retrospective single-center study of invasively ventilated patients

Abdelrahman Mahmoud M. Senosy[1], Charalampos Pierrakos[2], Ary Serpa Neto[3], Marcus J. Schultz[4]

Table 1S - Alternative formulas for mechanical power estimation

Name	Units	Formula
MP_{PC}(1)	J/minute	0.098 * VT (L) * RR * (PEEP + ΔP)
MP_{NORM}(2)	J/minute/kg per PBW	(0.098 * V_T (L) * RR * (Pmax – ½ * ΔP)/ PBW
MP_{CRS}(3)	J/minute × cmH_2O/mL	(0.098 * V_T (L) * RR * (Pmax – ½ * ΔP)/C_{RS}) * 10^3
$MP_{CRS\text{-}NORM}$(4)	J/minute x cmH_20/mL/kg per PBW	(0.098 * V_T (L) * RR * (Pmax – ½ * ΔP)/C_{RS} * PBW)*10^3
Dynamic power(5)	J/minute	0.098 * V_T (L) * RR * ΔP
Costa index(6)	No units	4*ΔP+RR

MP_{PC} - mechanical power adapted for pressure control ventilation mode; V_T - tidal volume; RR - respiratory rate; PEEP - positive end expiratory pressure; ΔP - airway pressure above PEEP; MP_{NORM} - mechanical power normalized to predicted body weight; PBW - predicted body weight; MP_{CRS} - mechanical power normalized to respiratory system compliance; $MP_{CRS\text{-}NORM}$ - mechanical power normalized to respiratory system compliance and to predicted body weight.

1. Chiumello D, Gotti M, Guanziroli M, Formenti P, Umbrello M, Pasticci I, et al. Bedside calculation of mechanical power during volume- and pressure-controlled mechanical ventilation. Crit Care. 2020;24(1):417.
2. Zhang Z, Zheng B, Liu N, Ge H, Hong Y. Mechanical power normalized to predicted body weight as a predictor of mortality in patients with acute respiratory distress syndrome. Intensive Care Med. 2019;45(6):856-64.
3. Yan Y, Du Z, Chen H, Liu S, Chen X, Li X, et al. The relationship between mechanical power normalized to dynamic lung compliance and weaning outcomes in mechanically ventilated patients. PLoS One. 2024;19(8):e0306116.
4. Deans KJ, Minneci PC, Suffredini AF, Danner RL, Hoffman WD, Ciu X, et al. Randomization in clinical trials of titrated therapies: unintended consequences of using fixed treatment protocols. Crit Care Med. 2007;35(6):1509-16.
5. Schaefer MS, Loring SH, Talmor D, Baedorf-Kassis EN. Comparison of mechanical power estimations in mechanically ventilated patients with ARDS: a secondary data analysis from the EPVent study. Intensive Care Med. 2021;47(1):130-2.
6. Costa EL, Slutsky AS, Brochard LJ, Brower R, Serpa-Neto A, Cavalcanti AB, et al. Ventilatory variables and mechanical power in patients with acute respiratory distress syndrome. Am J Respir Crit Care Med. 2021;204(3):303-11.

This is an open access article under the CC BY license (https://creativecommons.org/licenses/by/4.0/).

Table 2S - Ventilatory parameters of the patients shown for both the total population and subgroups categorized by low and high respiratory system compliance, with a cut-off value of 36mL/cmH$_2$O

Variables<br>Total population	2003 - 2009			2010 - 2016		
	Low C_{RS}	High C_{RS}	Total population	Low C_{RS}	High C_{RS}	
Tidal volume per PBW (mL/kg)	6.9 (6.1 - 7.8)	6.6 (5.9 - 7.4)	7.2 (6.5 - 8.2)	6.3 (5.5 - 7.4)	5.7 (5.1 - 6.5)	6.9 (6.1 - 8.1)
Number of patients	2,233	1,489	744	2,299	1,274	1,025
Tidal volume per PBW $<$ 6mL/kg, (%)	442 (20)	388 (26)	55 (7)	905 (39)	757 (59)	148 (14)
Tidal volume per PBW 6 - 8mL/kg, (%)	1316 (59)	922 (61)	394 (53)	1016 (44)	486 (38)	530 (52)
Tidal volume per PBW 8.1 - 10mL/kg, (%)	422 (18)	172 (11)	250 (34)	267 (12)	30 (2)	237 (24)
Tidal volume per PBW 10.1 - 12mL/kg, (%)	46 (2)	6 (1)	40 (5)	87 (4)	1 (1)	86 (8)
Tidal volume per PBW $>$ 12mL/kg (%)	7 (1)	1 (1)	6 (1)	24 (1)	0 (0)	24 (2)
Respiratory rate (breaths/min)	17.1 (15.2 - 19.5)	18.2 (15.7 - 20.8)	15.8 (15.1 - 17.5)	18.2 (15.7 - 20.7)	19.3 (16.7 - 22.5)	17.2 (15.1 - 19.5)
Number of patients	2,504	1,335	1,169	2,283	1,041	1,242
Respiratory rate $\leq$ 15 breaths/minute, (%)	705 (28)	258 (19)	447 (38)	465 (20)	129 (12)	336 (27)
Respiratory rate 16 - 20 breaths/minute, (%)	1281 (51)	651 (49)	630 (54)	1130 (49)	477 (46)	653 (53)
Respiratory rate 21 - 25 breaths/minute, (%)	440 (18)	353 (26)	87 (7)	542 (24)	318 (30)	224 (18)
Respiratory rate $>$ 25 breaths/minute, (%)	78 (3)	73 (6)	5 (1)	146 (7)	117 (12)	29 (2)
Driving pressure (cmH$_2$O)	13.5 (11.0 - 16.6)	16.2 (14.1 - 18.7)	11.1 (9.2 - 12.5)	11.2 (8.5 - 14.5)	14.6 (12.6 - 17.1)	8.9 (6.5 - 10.6)
Number of patients	2,536	1,339	1,197	2,341	1,055	1,286
Driving pressure $<$ 10cmH$_2$O, (%)	473 (19)	18 (1)	455 (38)	892 (38)	37 (3)	855 (66)
Driving pressure 10 - 14.9cmH$_2$O, (%)	1149 (45)	468 (35)	681 (57)	964 (41)	552 (52)	412 (33)
Driving pressure 15 - 20cmH$_2$O, (%)	703 (28)	642 (48)	61 (5)	390 (17)	371 (36)	19 (1)
Driving pressure $>$ 20cmH$_2$O, (%)	211 (8)	211 (16)	0 (0)	95 (4)	95 (9)	0 (0)
Mechanical power (J/minute)	12.1 (8.7 - 16.7)	13.4 (9.7 - 19.1)	10.5 (8.1 - 14.5)	11.2 (8.5 - 14.6)	11.7 (8.2 - 16.3)	9.7 (7.2 - 13.3)
Number of patients	2,504	1,335	1,169	2,283	1,041	1,242
Mechanical power $<$ 12 J/minute, (%)	1221 (49)	529 (40)	692 (59)	1380 (60)	549 (52)	831 (67)
Mechanical power 12 - 16.9 J/minute, (%)	683 (27)	362 (27)	321 (27)	506 (22)	256 (25)	250 (20)
Mechanical power 17 - 22 J/minute, (%)	326 (13)	215 (16)	111 (10)	236 (10)	134 (13)	102 (8)
Mechanical power $>$ 22 J/minute, (%)	274 (11)	229 (17)	45 (4)	161 (8)	102 (10)	59 (5)

C_{RS} - respiratory system compliance; PBW - predicted body weight. Results are presented as medians with interquartile ranges or percentages where appropriate.

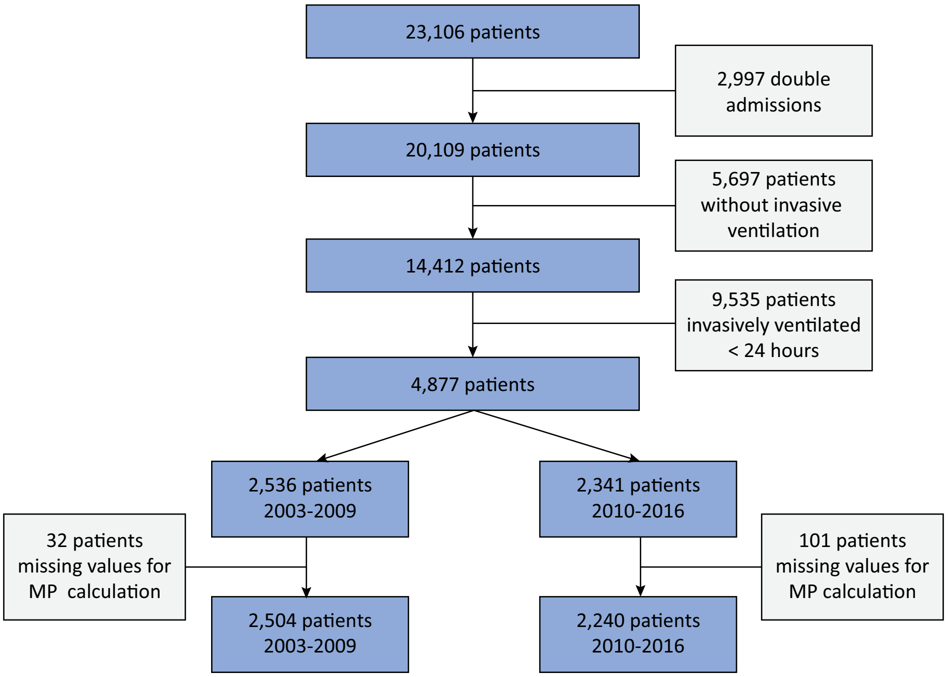

MP - mechanical power.

Figure 1S - The flowchart of the patients.

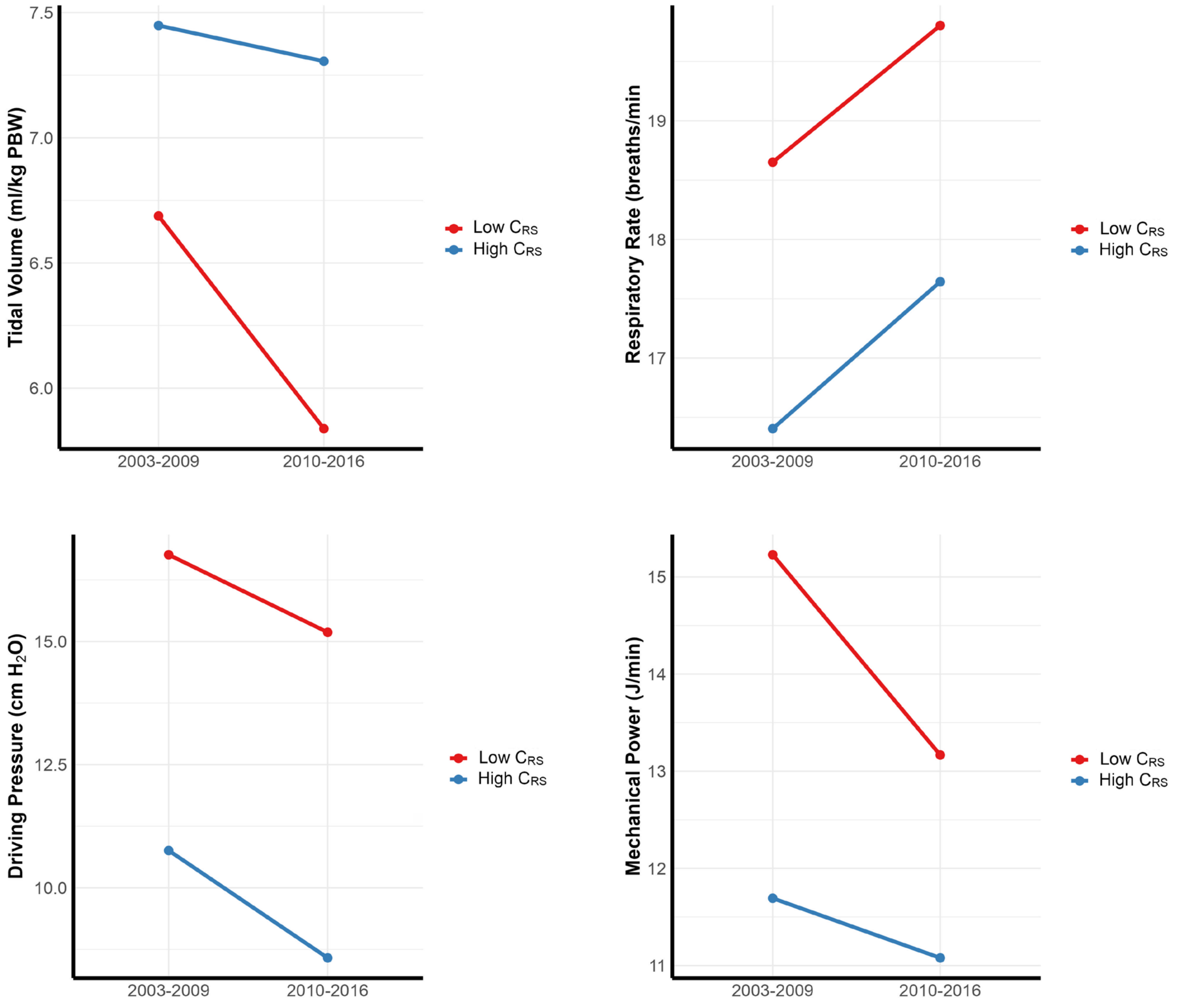

PBW - predicted body weight; C_{RS} - respiratory system compliance

Figure 2S - Interaction plots comparing the mean tidal volume, respiratory rate, driving pressure, and mechanical power across two cohort periods, 2003 - 2009 and 2010 - 2016, stratified by respiratory system compliance category.

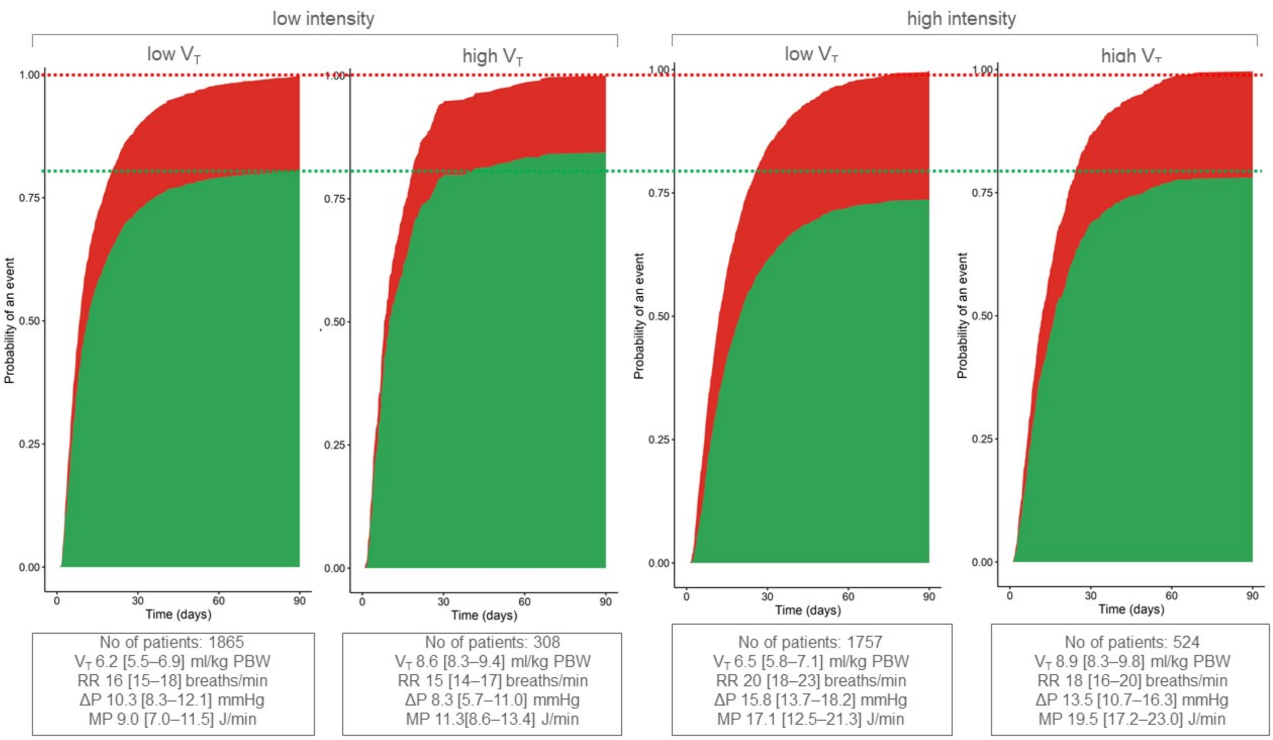

V_T - tidal volume; RR - respiratory rate; ΔP - driving pressure; MP - mechanical power.

Figure 3S - Cumulative incidence of intensive care unit discharge and mortality within 90 days by ventilation intensity and tidal volume per predicted body weight.

The x–axis shows days since intensive care unit admission, and the y–axis represents the probability of intensive care unit discharge (green) or death (red). The left panels represent low-intensity ventilation and the right panels high-intensity ventilation with low (tidal volume < 8mL/kg predicted body weight) and higher tidal volumes (tidal volume ≥ 8mL/kg predicted body weight). Below each panel are key ventilatory parameters: tidal volume, respiratory rate, driving pressure, and mechanical power. The red line marks the incidence of any event (intensive care unit discharge or death), while the green line marks the highest intensive care unit discharge in patients receiving low-intensity and low tidal volume ventilation.

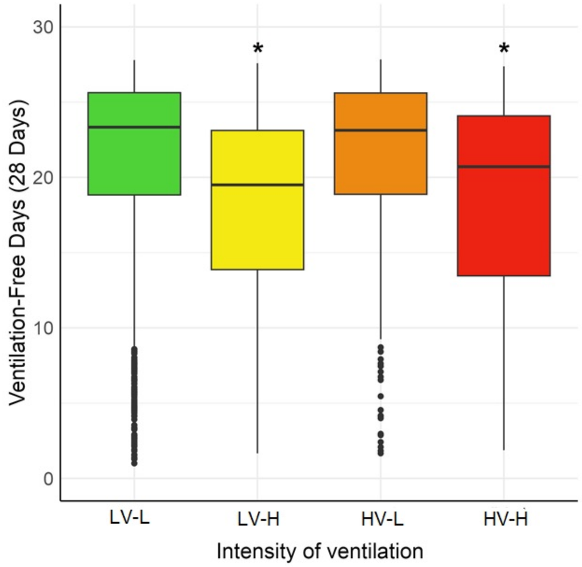

LV-L - low tidal volume ventilation; LV-H - low tidal volume ventilation at high-intensity; HV-L - high tidal volume ventilation at low intensity; HV-H - high tidal volume at high-intensity.

Figure 4S - Ventilation-free days within 28 days of intensive care unit hospitalization in survivals according to the types of ventilation: lung-protective ventilation (lung-protective ventilation, green color), low tidal volume ventilation (tidal volume < 8mL/kg predicted body weight) but high driving pressure and/or mechanical power (LV-H, yellow color), high V_T ventilation but low driving pressure and mechanical power (HV-L, orange color), and high tidal volume ventilation and high driving pressure and/or mechanical power (HV-H, red color). * p value < 0.05 compared with lung-protective ventilation.

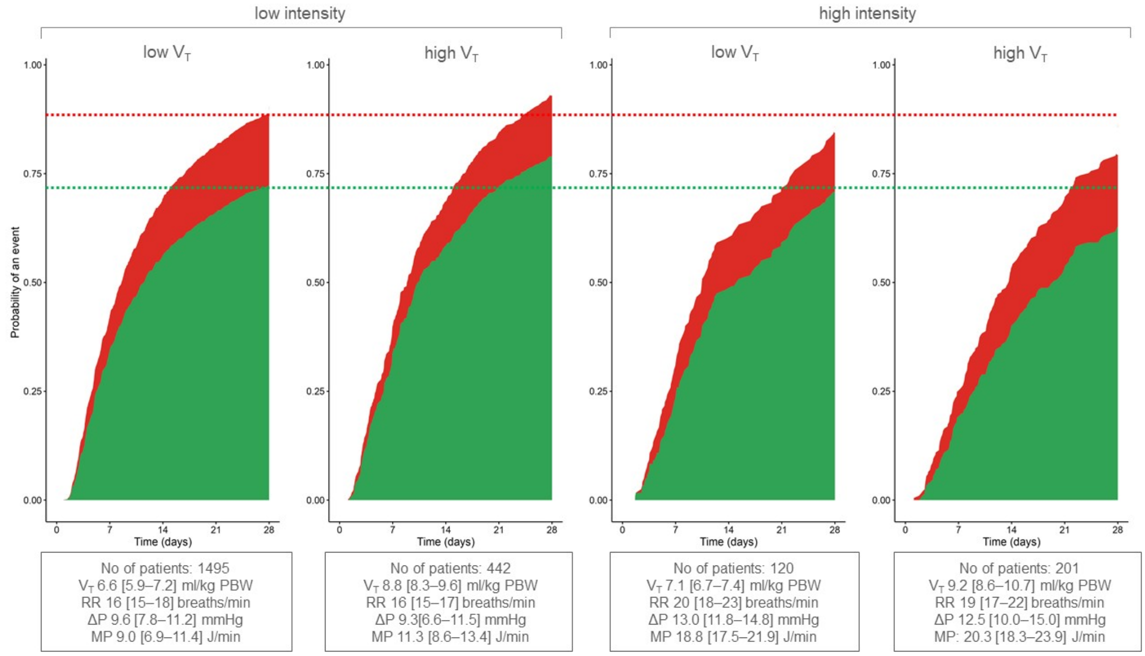

V_T - tidal volume; PBW - predicted body weight RR - respiratory rate; ΔP - driving pressure; MP - mechanical power.

Figure 5S - Cumulative incidence of intensive care unit discharge and mortality within 28 days, in patients with high compliance, by ventilation intensity and tidal volume per predicted body weight.

The x–axis shows days since intensive care unit admission, and the y–axis represents the probability of intensive care unit discharge (green) or death (red). The left panels represent low-intensity ventilation and the right panels high-intensity ventilation with low (tidal volume < 8mL/kg predicted body weight) and higher tidal volumes (tidal volume ≥ 8mL/kg predicted body weight). Below each panel are key ventilatory parameters: tidal volume, respiratory rate, driving pressure, and mechanical power. The red line marks the highest incidence of any event (intensive care unit discharge or death), while the green line marks the highest intensive care unit discharge in patients receiving low-intensity and low tidal volume ventilation.

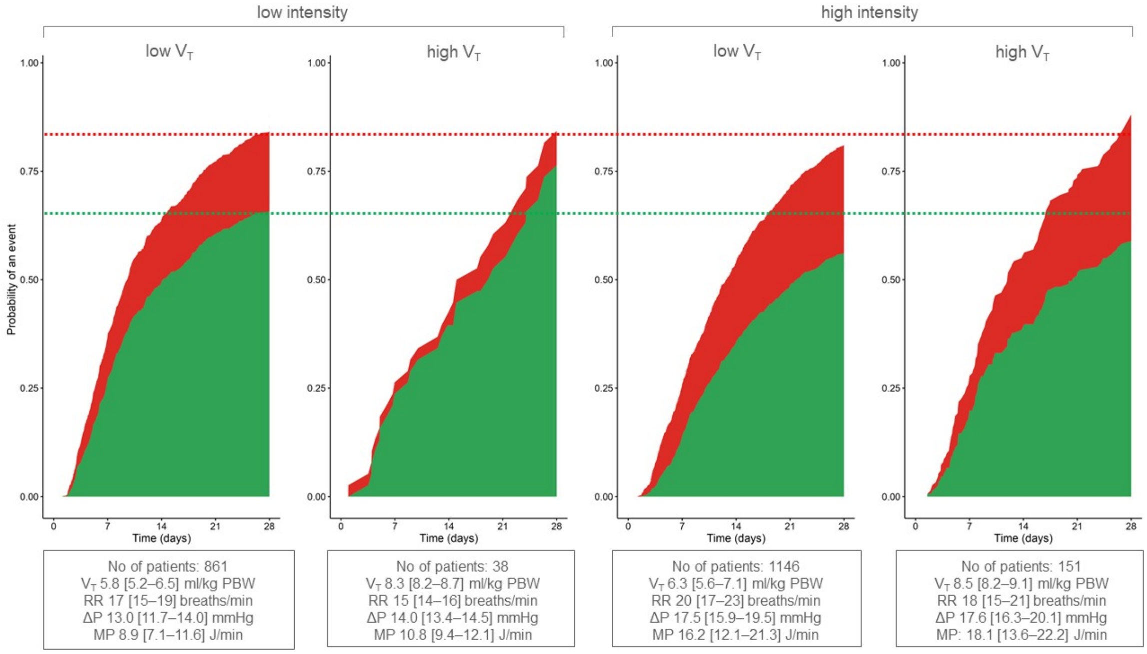

V_T - tidal volume; PBW - predicted body weight RR - respiratory rate; ΔP - driving pressure; MP - mechanical power.

Figure 6S - Cumulative incidence of intensive care unit discharge and mortality within 28 days, in patients with *low compliance*, by ventilation intensity and tidal volume per predicted body weight.

The x–axis shows days since intensive care unit admission, and the y–axis represents the probability of intensive care unit discharge (green) or death (red). The left panels represent low-intensity ventilation and the right panels high-intensity ventilation with low (tidal volume < 8mL/kg predicted body weight) and higher tidal volumes (tidal volume ≥ 8mL/kg predicted body weight). Below each panel are key ventilatory parameters: tidal volume, respiratory rate, driving pressure, and mechanical power. The red line marks the highest incidence of any event (intensive care unit discharge or death), while the green line marks the highest intensive care unit discharge in patients receiving low-intensity and low tidal volume ventilation.

Post hoc analysis repeating the analysis in medical and surgical patients separately

From 2003 - 2009 to 2010 - 2016, median tidal volume (V_T) decreased (mean difference (MD) of 0.71mL/kg predicted body weight (PBW); p < 0.01 and 0.44mL/kg PBW; p < 0.01]) and median respiratory rate (RR) increased (1.0 breath/minute; p < 0.01 and 1.2 breath/minute; p < 0.01) in medical and surgical patients (Table 4S). The percentage of patients that received low V_T ventilation (LTVV) increased from 79% to 81%, and from 78% to 83% in medical and surgical patients (Table 3S). The secular changes in V_T and RR were associated with a decrease in median mechanical power (MP), from 13.0 (9.2 - 18.3) J/minute to 11.3 (8.1 - 16.1) J/minute in medical patients (MD -1.7 [-1.9 to -1.5] J/minute; p < 0.01), and from 11.3 (8.3 - 15.0) J/minute to 9.7 (7.1 - 13.1) J/minute in surgical patients (MD-1.5 [-1.7 to -1.4] J/minute; p < 0.01) (Figures 7S and 8S). The percentage of patients with MP < 17 J/minute increased from 74% to 80% in medical patients, and from 83% to 89% in surgical patients.

Table 3S - Characteristics of patients from 2003 - 2009 and 2010 - 2016, presented for the overall population and separately for medical and surgical admissions

Baseline characteristics Total population	2003-2009			2010-2016		
	Medical	Surgical	Total population	Medical	Surgical	
Number of patients	2,536	1,298	1,238	2,341	1,440	901
Female (gender)	883 (35)	471 (38)	375 (34)	805 (34)	485 (34)	320 (35)
Age of patients	65 (55 - 75)	64 (54 - 74)	64 (54 - 74)	65 (55 - 75)	64 (54 - 74)	64 (54 - 74)
Weight (kg)	75 (65 - 85)	74 (64 - 84)	75 (74 - 84)	75 (65 - 85)	74 (64 - 84)	84 (74 - 84)
Height (cm)	175 (165 - 185)	174 (164 - 184)	174 (174 - 184)	175 (165 - 185)	174 (164 - 184)	174 (174 - 184)
Predicted body weight (kg)	70 (61 - 79)	70 (61 - 79)	70 (65 - 79)	70 (61 - 79)	70 (61 - 79)	70 (61 - 79)
Low tidal volume ventilation (%)	1,758 (78)	898 (79)	860 (78)	1,921 (82)	1,173 (81)	748 (83)
Admission Type						
Surgical elective (%)	696 (27)	0 (0)	696 (26)	387 (17)	0 (0)	387 (20)
Surgical urgency (%)	542 (21)	0 (0)	542 (22)	514 (22)	0 (0)	514 (25)
Medical (%)	1,298 (51)	1,175 (53)	0(0)	1,440 (61)	1,439 (66)	0 (0)
Need for support the first 24 hours						
Vasopressors (%)	2,283 (90)	1,152 (88)	1,131 (89)	2036 (90)	1,208 (95)	828 (91)
Renal replacement therapy (%)	411 (16)	234 (18)	177 (14)	336 (15)	214 (17)	122 (13)
Initial diagnosis						
Sepsis (%)	464 (18)	247 (19)	217 (17)	390 (17)	288 (20)	102 (11)
Cardiovascular disease (%)	708 (28)	403 (31)	305 (25)	638 (27)	418 (29)	220 (24)
Respiratory condition (%)	265 (10)	142 (11)	123 (10)	338 (14)	216 (15)	122 (14)
Neurological condition (%)	222 (9)	91 (7)	131 (11)	304 (13)	100 (7)	204 (23)
Trauma (%)	66 (3)	25 (2)	41 (3)	170 (7)	58 (4)	112 (12)
Other (%)	811 (32)	390 (30)	421 (34)	501 (21)	360 (25)	141 (16)
Severity of disease						
APACHE II score	21 (17 - 26)	22 (18 - 27)	21 (17 - 25)	23 (19 - 28)	24 (19 - 30)	21 (17 - 26)
SOFA score	9 (7 - 11)	9 (7 - 11)	9 (7 - 11)	9 (7 - 11)	9 (7 - 12)	9 (7 - 11)
Vital signs						
PaO_2/FiO_2 (mmHg)	147 (96 - 207	144 (85 - 200)	152 (97 - 214)	145 (97 - 210)	134 (94 - 191)	166 (108 - 232)
Heart rate (beats/minute)	106 (91 - 124)	109 (92 - 128)	104 (90 - 120)	116 (98 - 135)	117 (98 - 138)	119 (103 - 137)
Mean arterial pressure (mmHg)	60 (53 - 66)	59 (53 - 65)	60 (54 - 67)	52 (38 - 61)	51 (38 - 60)	50 (37 - 61)
Temperature (degree Celsius)	37 (36 - 37)	36 (35 - 37)	36 (35 - 37)	37 (36 - 37)	36 (35 - 37)	36 (35 - 37)

Continue...

...continuation

Laboratory data						
pH	7.29 (7.21 - 7.34)	7.33 (7.25 - 7.40)	7.37 (7.31 - 7.41)	7.27 (7.19 - 7.33)	7.32 (7.23 - 7.40)	7.36 (7.29 - 7.41)
$PaCO_2$ (mmHg)	39 (36 - 43)	39 (36 - 44)	39 (36 - 43)	41 (37 - 45)	41 (36 - 46)	41 (37 - 44)
CRP (mg/L)	63 (11 - 149)	59 (9 - 150)	62 (12 - 148)	55 (8 - 140)	60 (10 - 147)	45 (6 - 130)
Outcomes						
Ventilation free days	17 (2 - 23)	21 (14 - 24)	21 (10 - 25)	20 (4 - 25)	22 (16 - 25)	23 (12 - 26)
ICU mortality (%)	523 (21)	326 (25)	197 (15)	571 (24)	414 (29)	157 (18)

APACHE II - Acute Physiology and Chronic Health Evaluation II; SOFA - Sequential Organ Failure Assessment; PaO_2 - partial pressure of arterial oxygen; FiO_2 - fraction of inspired oxygen; $PaCO_2$ - partial pressure of arterial carbon dioxide; CRP - C-reactive protein; ICU - intensive care unit. Results are presented as medians with interquartile ranges or percentages where appropriate.

Table 4S - Ventilation parameters of patients from 2003 - 2009 and 2010 - 2016, presented for the overall population and separately for medical and surgical admissions

Variables	2003 - 2009			2010 - 2016		
	Total population	Medical	Surgical	Total population	Medical	Surgical
Compliance (mLcm H_2O)	34.9 (27.4 - 45.2)	33.4 (26.0 - 43.2)	36.5 (28.7 - 46.6)	38.1 (28.1 - 56.6)	35.3 (26.2 - 50.0)	42 (31 - 63)
Tidal volume (mL)	456 (422 - 536)	479 (422 - 535)	473 (421 - 537)	435 (378 - 511)	426 (374 - 493)	448 (481 - 523)
Tidal volume per PBW (mL/kg)	6.9 (6.1 - 7.8)	6.9 (6.2 - 7.7)	6.9 (6.1 - 7.9)	6.3 (5.5 - 7.4)	6.2 (5.4 - 7.2)	6.5 (5.6 - 7.5)
PEEP (cmH_2O)	8.5 (6.2 - 10.5)	8.6 (6.5 - 11.1)	8.1 (5.7 - 10.1)	7.9 (5.8 - 9.9)	8.2 (6.3 - 10.5)	7.2 (5.2 - 9.2)
Respiratory rate (breaths/minute)	17.1 (15.2 - 19.5)	17.5 (15.3 - 20.1)	16.2 (14.8 - 17.3)	18.2 (15.7 - 20.7)	18.5 (16.0 - 21.6)	17.5 (15.5 - 20.0)
Driving pressure (cmH_2O)	13.5 (11.0 - 16.6)	14.1 (11.5 - 17.3)	13.0 (10.6 - 15.7)	11.2 (8.5 - 14.5)	13.8 (11.8 - 16.0)	10.5 (7.8 - 13.3)
MP_{dyn} (J/minute)	12.1 (8.7 - 16.7)	13.0 (9.2 - 18.3)	11.3 (8.3 - 15.0)	11.2 (8.5 - 14.6)	11.3 (8.1 - 16.1)	9.7 (7.1 - 13.1)
MP_{PC}	17.4 (12.8 - 23.9)	19.1 (13.5 - 26.2)	16.3 (12.3 - 21.5)	14.8 (10.8 - 20.7)	16.1 (11.6 - 22.8)	13.9 (10.2 - 18.3)
MP_{NORM} (J/minute/kg per PBW)	177 (130 - 245)	191 (134 - 264)	166 (125 - 225)	153 (110 - 214)	164 (118 - 237)	142 (105 - 192)
MP_{CRS} (J/minute $\times$ cmH_2O/mL)	341 (218 - 552)	394 (238 - 631)	306 (202 - 467)	268 (158 - 447)	311 (189 - 537)	219 (132 - 372)
$MP_{CRS\text{-}NORM}$ (J/minute x cmH_2O/mL/kg per PBW)	5.1 (3.1 - 8.2)	5.6 (3.4 - 9.4)	4.4 (2.9 - 6.7)	3.8 (2.2 - 6.6)	4.5 (2.6 - 7.9)	3.3 (1.9 - 5.3)
Dynamic power (J/minute)	10.7 (7.8 - 14.5)	11.7 (8.3 - 15.7)	9.9 (7.5 - 13.2)	8.6 (6.1 - 12.1)	9.4 (6.6 - 13.2)	6.6 (4.5 - 9.2)
Costa index	71 (60 - 85)	75 (62 - 89)	69 (58 - 81)	63 (52 - 77)	67 (55 - 82)	59 (48 - 72)
Type of ventilation						
Number of patients	2,209	1,116	1,093	2,245	1,376	869
Low tidal volume - Low intensity (%)	769 (35)	460 (41)	309 (28)	1096 (49)	762 (55)	334 (38)
Low tidal volume - High intensity (%)	974 (44)	433 (39)	541 (49)	783 (35)	384 (28)	399 (46)
High tidal volume - Low intensity (%)	149 (7)	94 (9)	55 (5)	159 (7)	127 (9)	32 (4)
High tidal volume - High intensity (%)	317 (14)	129 (11)	188 (17)	207 (9)	103 (8)	104 (12)

PBW - predicted body weight; PEEP - positive end-expiratory pressure MP_{dyn} - dynamic mechanical power; MP_{PC} - mechanical power adapted for pressure control ventilation mode; MP_{NORM} - dynamic mechanical power normalized to predicted body weight; MP_{CRS} - dynamic mechanical power normalized to respiratory system compliance; $MP_{CRS\text{-}NORM}$: - dynamic mechanical power normalized to respiratory system compliance and to predicted body weight. Results are presented as medians with interquartile ranges or percentages where appropriate.

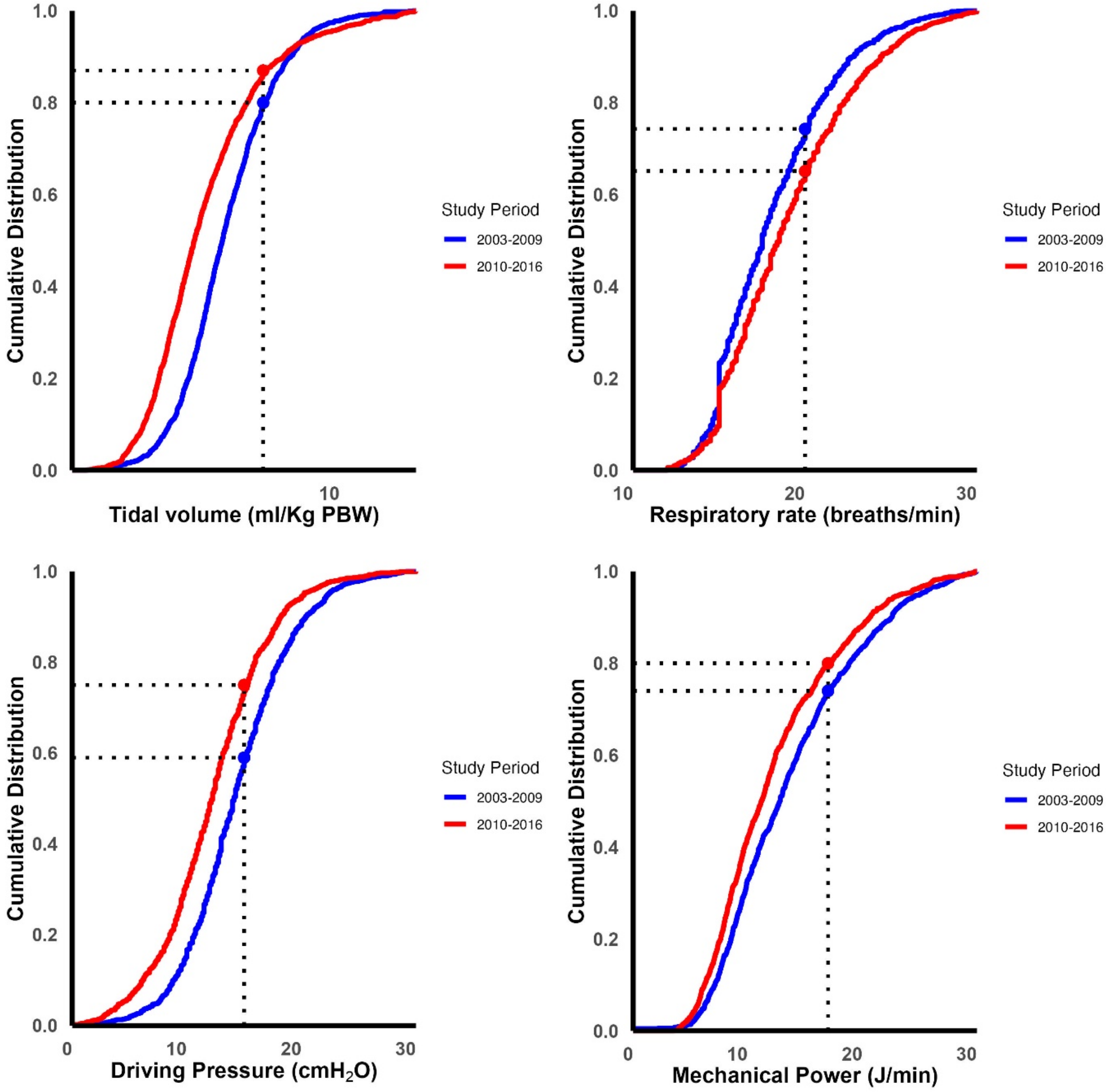

PBW - predicted body weight.

Figure 7S - Cumulative distribution curves for tidal volume, respiratory rate, driving pressure and mechanical power of ventilation comparing two study periods, from 2003 to 2009 and from 2010 to 2016 for patients admitted to intensive care unit with medical diagnosis.

The dotted lines indicate the cutoff values used to define lung–protective ventilation strategies, for tidal volume (8mL/kg of predicted body weight), driving pressure (15cmH_2O), respiratory rate (20 breaths/minute), and mechanical power of ventilation (17J/minute).

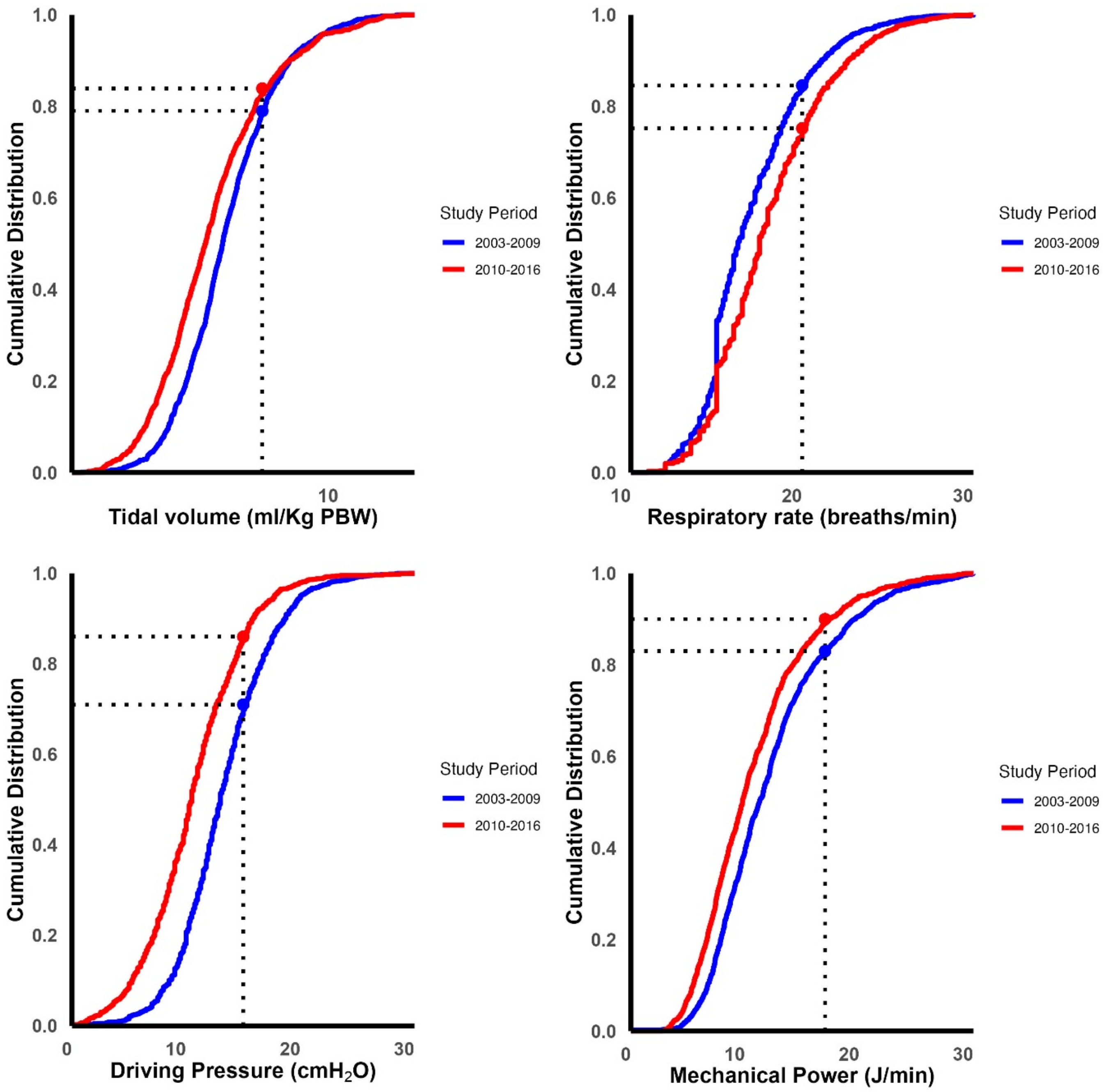

PBW - predicted body weight.

Figure 8S - Cumulative distribution curves for tidal volume, respiratory rate, driving pressure and mechanical power of ventilation comparing two study periods, from 2003 to 2009 and from 2010 to 2016 for patients admitted to intensive care unit with surgical diagnosis.

The dotted lines indicate the cutoff values used to define lung–protective ventilation strategies, for tidal volume (8mL/kg of predicted body weight), driving pressure (15cmH_2O), respiratory rate (20 breaths/minute), and mechanical power of ventilation (17 J/minute).
